# An update of pitfalls in prostate mpMRI: a practical approach through the lens of PI-RADS v. 2 guidelines

**DOI:** 10.1007/s13244-017-0578-x

**Published:** 2017-10-23

**Authors:** Valeria Panebianco, Francesco Giganti, Yu Xuan Kitzing, François Cornud, Riccardo Campa, Gianluca De Rubeis, Antonio Ciardi, Carlo Catalano, Geert Villeirs

**Affiliations:** 1 Department of Radiological Sciences, Oncology & Pathology, Sapienza, University of Rome, V.le Regina Elena, 324 00161 Rome, Italy; 2 Department of Radiology, University College London Hospital NHS Foundation Trust, London, UK; 3 Department of Radiology, Cambridge University Hospitals NHS Foundation Trust Addenbrooke’s Hospital, Cambridge, UK; 4 Department of Radiology, Hôpital Cochin, Paris Descartes University, Sorbonne Paris Cité, Paris, France; 5 Department of Radiology, Ghent University Hospital, Ghent, Belgium

**Keywords:** Prostate, Prostatic cancer, Magnetic resonance imaging, Diagnosis, Pitfalls

## Abstract

**Objectives:**

The aim of the current report is to provide an update in the imaging interpretation of prostate cancer on multiparametric magnetic resonance imaging (mpMRI), with a special focus on how to discriminate pathological tissue from the most common pitfalls that may be encountered during daily clinical practice using the Prostate Imaging Reporting and Data System (PI-RADS) version 2 guidelines.

**Methods:**

All the cases that are shown in this pictorial review comply with the European Society of Urogenital Radiology (ESUR) guidelines for technical mpMRI requirements.

**Results:**

Despite the standardised manner to report mpMRI (PI-RADS v. 2), some para-physiologic appearances of the prostate can mimic cancer. As such, it is crucial to be aware of these pitfalls, in order to avoid the under/overestimation of prostate cancer.

**Conclusions:**

A detailed knowledge of normal and abnormal findings in mpMRI of the prostate is pivotal for an accurate management of the wide spectrum of clinical scenarios that radiologists may encounter during their daily practice.

***Teaching Points*:**

• *Some para-physiologic appearances of the prostate may mimic cancer.*

• *Knowledge of normal and abnormal findings in prostate mpMRI is pivotal.*

• *Any radiologist involved in prostate mpMRI reporting should be aware of pitfalls.*

## Introduction

High-quality multi-parametric Magnetic Resonance Imaging (mpMRI) has become an important tool in the diagnosis, characterisation and treatment planning of prostate cancer (PCa) [1–3]. Specifically, mpMRI involves T2-weighted anatomical images combined with functional imaging methods such as dynamic Contrast Enhanced Imaging (DCE), Diffusion-Weighted Imaging (DWI) and/or Spectroscopy, if necessary. All these sequences have a limited accuracy, when considered individually, but their association has shown a greater performance in the assessment of PCa [4].

**Table 1:** Wide spectrum of pitfalls and different classifications

Pitfall	Pitfall vs Pitfall
Anatomic 1. Hypertrophic anterior fibromuscular stroma Benign conditions 1. *Moustache sign* (small bilateral BPH nodules against the PZ) 2. *Moustache-like sign* (larger adenoma against the PZ) 3. *Teardrop sign* (median posterior compressed central zone) 4. *Teardrop-like sign* (Protruding BPH above the verumontanum) 5. Ectopic BPH nodule 6. Haemorrhage 7. Calcifications Overestimation 1. Periprostatic venous plexus 2. Neurovascular bundle 3. Prostatitis Mispositioned endorectal coil	I. PCa in moustache sign II. PCa in median posterior change (compressed central zone and BPH proliferation) in reversed teardrop III. Ectopic BPH nodule vs abscess

BPH = benign prostatic hyperplasia; PZ = peripheral zone; PCa = prostate cancer

However, inter-reader variability represents a real limitation for prostate mpMRI, and expertise in reporting is crucial to improve cancer detection and staging accuracy. Therefore, it is important to validate the current protocols [5]. In 2012, the European Society of Urogenital Radiology (ESUR) set up an expert panel to develop a standardised system for prostate mpMRI interpretation and reporting, under the name of Prostate Imaging Reporting and Data System (PI-RADS) [6]. In 2015 a revision of this classification led to the publication of PI-RADS v. 2 [7], with the aim to promote a global standardisation of these guidelines, and to reduce the variability in the acquisition, interpretation and reporting of prostate mpMRI. A detailed explanation of PI-RADS v. 2 is beyond the aim of this report, but it is worth summarising the main points of these guidelines: (1) DWI is the dominant sequence in the peripheral zone (PZ); (2) T2-weighted imaging (T2-WI) is the dominant sequence in the transitional zone (TZ); (3) the role of DCE is secondary to DWI in the PZ; and (4) there is an overall 5-point (from 1 to 5) PI-RADS assessment (1: low probability – 5: very high probability of clinically significant cancer).

Nonetheless, the interpretation of mpMRI of the prostate can be challenging and new radiological skills are needed, especially when potential pitfalls (i.e., normal anatomic structures, benign conditions of the prostate or artefacts due to technical issues) might be erroneously interpreted as pathological conditions. Moreover, the PI-RADS v. 2 guidelines may be subject to some interpretation variability according to the radiologists’ individual experience, lowering the ability to distinguish pitfalls from true malignancy [8]. Awareness of these pitfalls is therefore fundamental.

Currently, there are only three reports (two from the United States and one from Europe) [9–11] addressing the mpMRI pitfalls in PCa. They have all suggested some strategies to assist the radiologists in avoiding misdiagnosis (and consequently mistreatment), but a systematic approach on how to tackle these aspects applying PI-RADS v. 2 has yet to be reported.

Therefore, the purpose of this article is to provide a practical approach for imaging interpretation in PCa, with a special focus on how to apply PI-RADS v. 2 to discriminate pathological conditions from the most common pitfalls that could be encountered during daily clinical practice. Of note, this report is based on a pragmatic consensus among a panel of different international radiologists highly experienced in mpMRI of the prostate (VP, FG, YXK, FC, GV).

## PITFALLS and PI-RADS v.2

For the sake of completeness, the cases shown in this pictorial report represent a cohort of men aged 47–79 years, with prior suspicion of PCa based on abnormal digital rectal examination, rise of prostate specific antigen (PSA) and/or family history of PCa. The exams have been acquired on a 3.0 T system (Discovery MR750, GE Healthcare, Waukesha, WI), by using a 32 multi-channel (or 8 multi-channel + endorectal coil) surface phased-array body coil.

Awareness of diagnostic pitfalls is important to avoid both false-positive and false-negative interpretations. A systematic approach to mpMRI of the prostate using PI-RADS v. 2 helps in the identification of PCa, but there still remains a wide spectrum of pitfalls. These can be broadly split into two main groups: (1) pitfalls related to clinical indications and (2) technical and physiological artefacts (Table 1).

## Pitfalls related to clinical indications

These pitfalls are related both to the anatomy of the prostate and to certain benign conditions of the gland, which can mimic the presence of cancer. The typical appearance of PCa in the PZ is a focal hypointense area on T2-WI; however, other benign conditions, such as prostatitis, fibro-muscular bands and post-biopsy haemorrhage can mimic this signal change, leading to potential misdiagnoses.

### Anatomic pattern

#### Hypertrophic anterior fibromuscular stroma

**Fig. 1:**
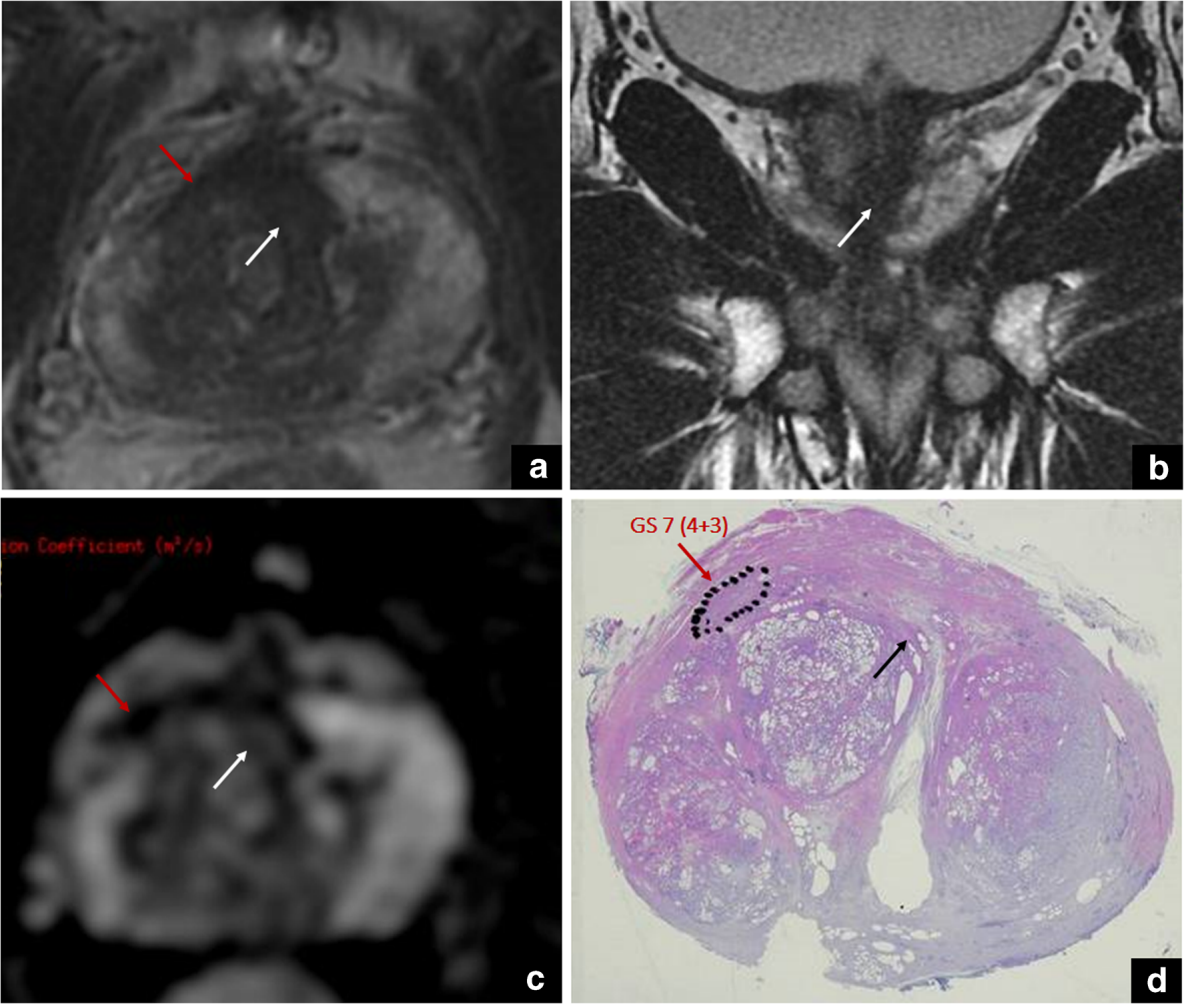
Hypertrophic anterior fibromuscular stroma vs cancer. Axial (**a**) and coronal (**b**) T2-weighted images that show an area of homogeneous low signal intensity with a lenticular shape (white arrows), and not significant restriction in the ADC map (**c**). The red arrows show a small focus of prostate cancer in the anterior right gland (**a**), corresponding to an area of restricted diffusion in the ADC map (**c**). These findings were confirmed at final histology, after radical prostatectomy (GS = Gleason score 4 + 3) (**d**)

This is related to the presence of muscle cells and connective tissue in the most anterior part of the gland, between the two lobes that constitute the TZ. This condition is characterised by an area of homogeneous, very low signal intensity on T2-WI, usually with lenticular shape (scored as 4/5 or 5/5 according to PI-RADS v. 2). However, hypertrophic anterior fibromuscular stroma does not commonly show enhancement on DCE nor significant restriction on DWI (score 1 or 2), even though low ADC values can be seen sometimes, due to pre-existing low T2-signal areas rather than a true restriction. Therefore, our suggestion is to use all the available planes, including the coronal and sagittal planes. An additional acquisition plan is of utmost importance, as it can confirm the continuity of the hypertrophic anterior fibromuscular stroma with the benign tissue. Such an approach, together with negative DWI and DCE findings, can help to rule out the presence of clinically significant PCa (Fig. 1).

#### Periprostatic neurovascular bundle

The periprostatic vascular (sometimes venous) plexus courses around the lateral margins of the prostate (i.e., very close to the capsule) and can show a congested appearance, particularly in men with prostatitis.

Sometimes it is difficult to separate the plexus from the PZ on mpMRI, due to focal low T2 signal intensity. This makes the use of PI-RADS v.2 mandatory.

**Fig. 2:**
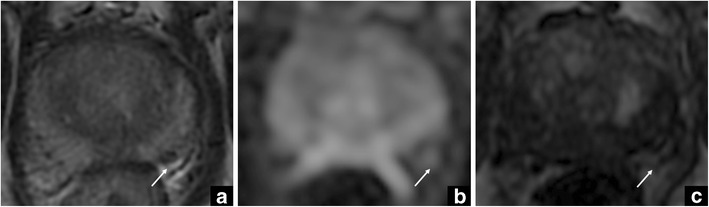
Periprostatic bundle. The arrow in the axial T2-weighted image (**a**) shows an area of intermediate to low-signal intensity in the right peripheral zone. This corresponds to mild, restricted diffusion on the echo-planar diffusion-weighted sequence (**b**), due to the slow speed of blood flow and focal enhancement on DCE imaging (**c**)

According to PI-RADS v. 2, the plexus can be scored as 3–4/5 on T2-WI, due to its mass-like appearance. Sometimes, this anatomical structure can show a mildly restricted diffusion (3/5) – only on Echo-planar sequence and not on the ADC map, due to the slow speed of blood flow - together with focal enhancement (+), and in continuity with the vessels, raising the suspicion of clinically significant PCa (mimicking T3a stage disease). In order to rule out the presence of a tumour, it is of paramount importance to take into account the appearance of the periprostatic plexus on T2-WI, as well as on delayed subtracted contrast-enhanced images (Fig. 2).

The neurovascular bundle courses along the posterolateral margin of the prostate, near the prostate capsule, at approximately a 5- and 7-o’clock position.

Similar to the periprostatic vascular plexus, the neurovascular bundle, when visible, can mimic the presence of a lesion in the PZ (mimicking T3a disease).

**Fig. 3:**
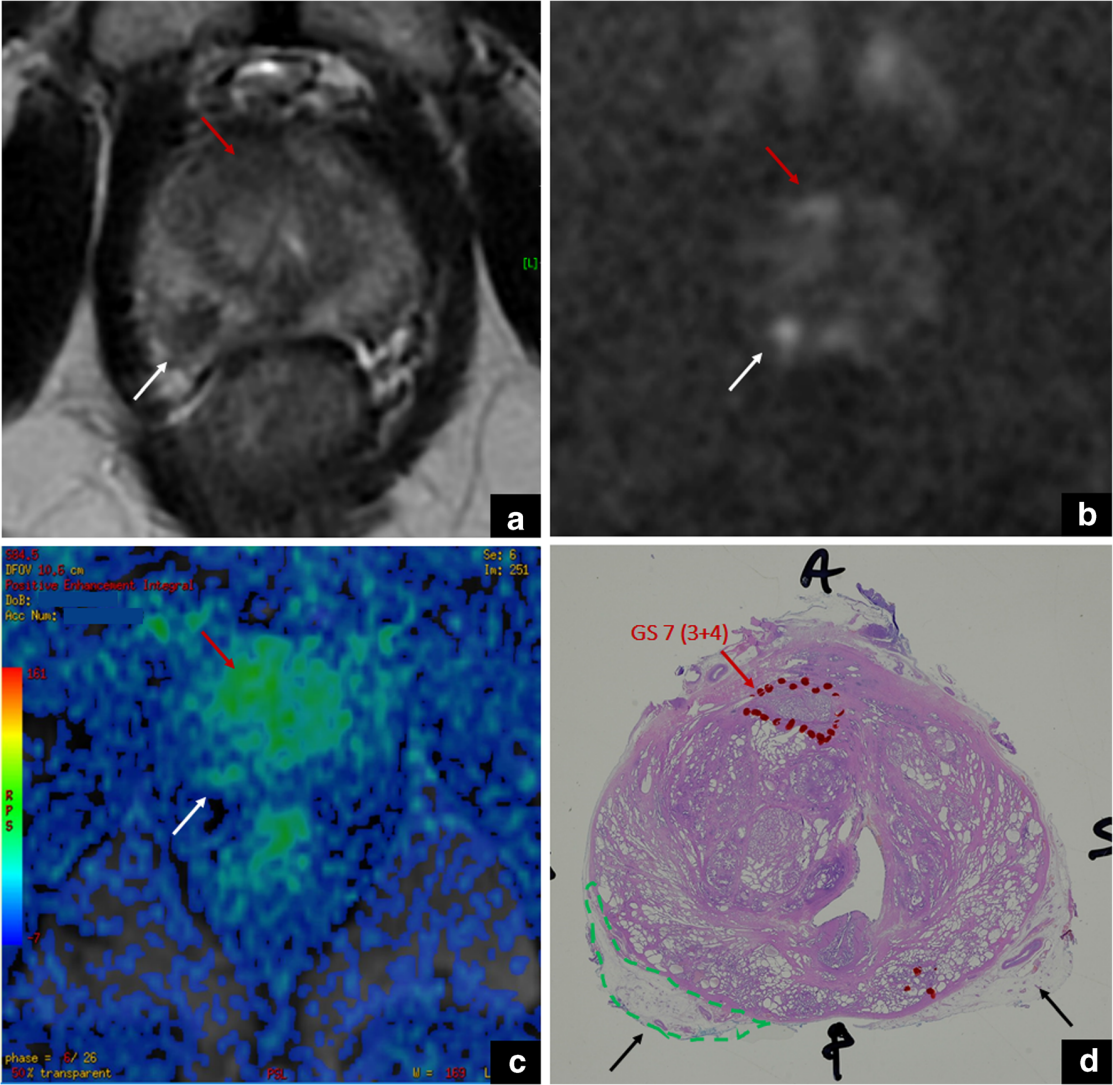
Neurovascular bundle vs cancer. The white arrows show the neurovascular bundle in the axial T2-weighted (**a**) and DW (**b**) images, and in the DCE map (**c**). The high signal intensity on DWI (i.e., restriction) on DWI is due to myelinated nerve fibres. The red arrows show a tumour in the right anterior gland. These findings were confirmed at final histology, after radical prostatectomy (GS = Gleason score 3 + 4) (**d**)

When difficult to discriminate from PZ, the neurovascular bundle can be scored as 4/5 on T2-WI according to PI-RADS v. 2, due to the very low signal intensity on this sequence. However, the neurovascular bundle can show restriction on DWI (4–5/5) due to signal from myelinated fibres and is characterised by a mild, enhancement pattern (+), tangent to the capsule. In this case the application of PI-RADS v. 2, and the knowledge of anatomy on DWI and DCE sequences is decisive to rule out the presence of PCa (Fig. 3).

In case of a tumour located adjacent to the periprostatic neurovascular bundle, the distinction might not be easy due to the absence of the adipose plane between them. At this regard, DCE sequences can be of help due to the different behaviour after contrast injection (early vs late/mild enhancement). Additionally, the coronal plane can help to localise the neurovascular bundle and the continuity of the capsule in another plan.

Moreover, mpMRI is increasingly being used in the decision-making pathway of PCa, to support the choice of a nerve-sparing approach when possible. In this regard, the use of diffusion-tensor imaging from mpMRI holds promise for the future [10].

### Benign conditions

#### Bilateral benign prostatic hyperplasia proliferation (*moustache sign*)

**Fig. 4:**
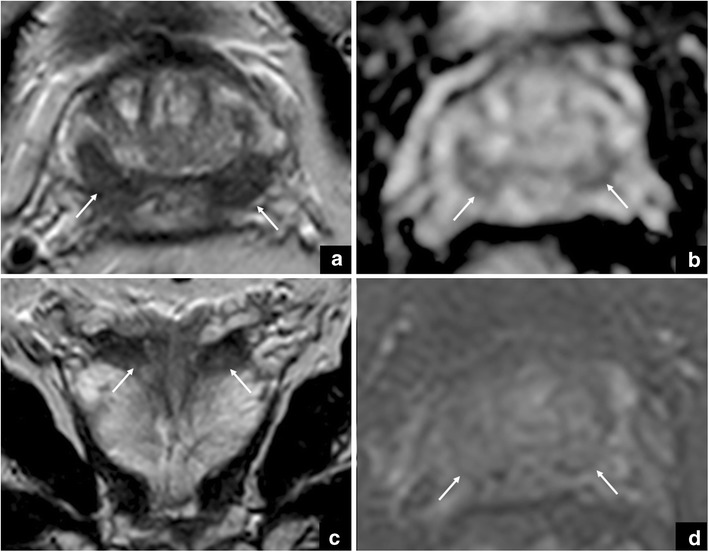
The arrows show two median, symmetric, bilateral areas of low signal intensity on axial (**a**) and coronal (**c**) T2-weighted imaging, with restricted diffusion in the ADC map (**b**) and diffuse enhancement on DCE imaging (**e**). This set of appearances has been called the “*moustache sign*”

**Fig. 5:**
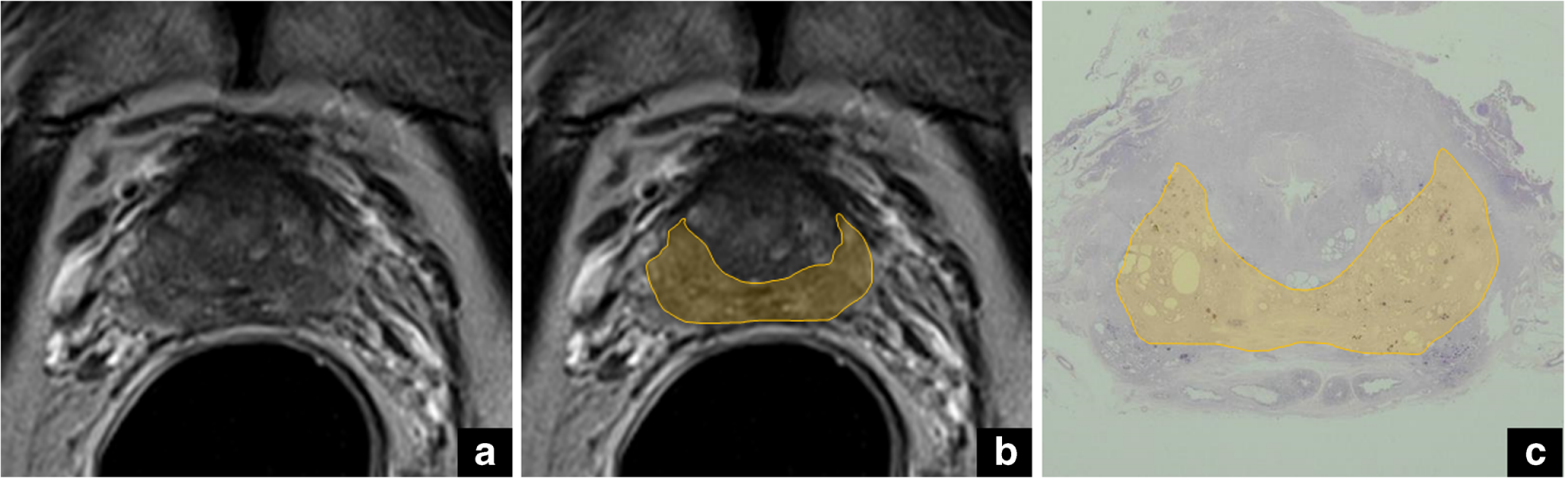
Axial T2-weighted image (**a**) of the posterior base. The yellow areas (**b**) correspond to the protrusion of a large adenoma in the peripheral zone (*moustache-sign like*), as confirmed at final histology, after radical prostatectomy (**c**)

The presence of median symmetric, bilateral areas of low signal intensity on T2-WI at the base/middle of the prostate on either side of the ejaculatory ducts can mimic cancer. This set of appearances has been called the “*moustache sign*”, and can be ascribed to two main conditions. The first is the compression of the central zone by small nodules of benign prostatic hyperplasia (BPH) against the PZ, and this typically occurs in the posterior portion of the prostate, at the base or mid-gland level (*moustache sign*) (Fig. 4). The second finding is related to the protrusion of a much larger adenoma in the PZ, and this also occurs in the posterior portion of the prostate, at the base (*moustache-sign like*) (Fig. 5).

On the contrary, foci of PCa generally show a more heterogeneous appearance, with ill-defined margins.

Histologically, the low signal intensity reflects respectively the compressed central gland, with hypertrophic tissue, and the different glandular pattern of BPH, with increased cellularity. In both cases, this sign typically appears as a symmetrical oval shape, with sharp margins and a homogeneous, low signal intensity.

These regions can also be characterised by restricted diffusion and homogeneous/positive enhancement. Protrusion of BPH nodules shows homogeneous enhancement, whereas the compressed central zone usually does not.

According to PI-RADS v. 2, a potential score for this pitfall could be 3–4/5 for T2-WI, 4/5 for DWI (in case of marked restriction of diffusion) and early enhancement (+) on DCE; such scenario would orient towards the presence of clinically significant PCa and might suggest biopsy. However, if we apply PI-RADS v. 2 after carefully considering other distinguishing features (e.g., the use of the coronal T2 weighted sequence, the presence of sharp margins and a symmetrical pattern), we can correctly score this condition as 2/5 both on T2-WI and DWI, excluding the presence of cancer (Figs. 4 and 5). It follows that an appropriate use of PI-RADS v. 2 guidelines is based on the knowledge and awareness of the anatomy related to BPH nodules in the central zone.

We also want to stress that the key feature to differentiate a pitfall from PCa is the *symmetry* of the finding. An asymmetrical area characterised by lower signal intensity on T2-WI with respect to the background (3) together with a marked, focal enhancement (+) and higher grade of restriction on DWI (4) suggests a suspicious lesion in this context.

**Fig. 6:**
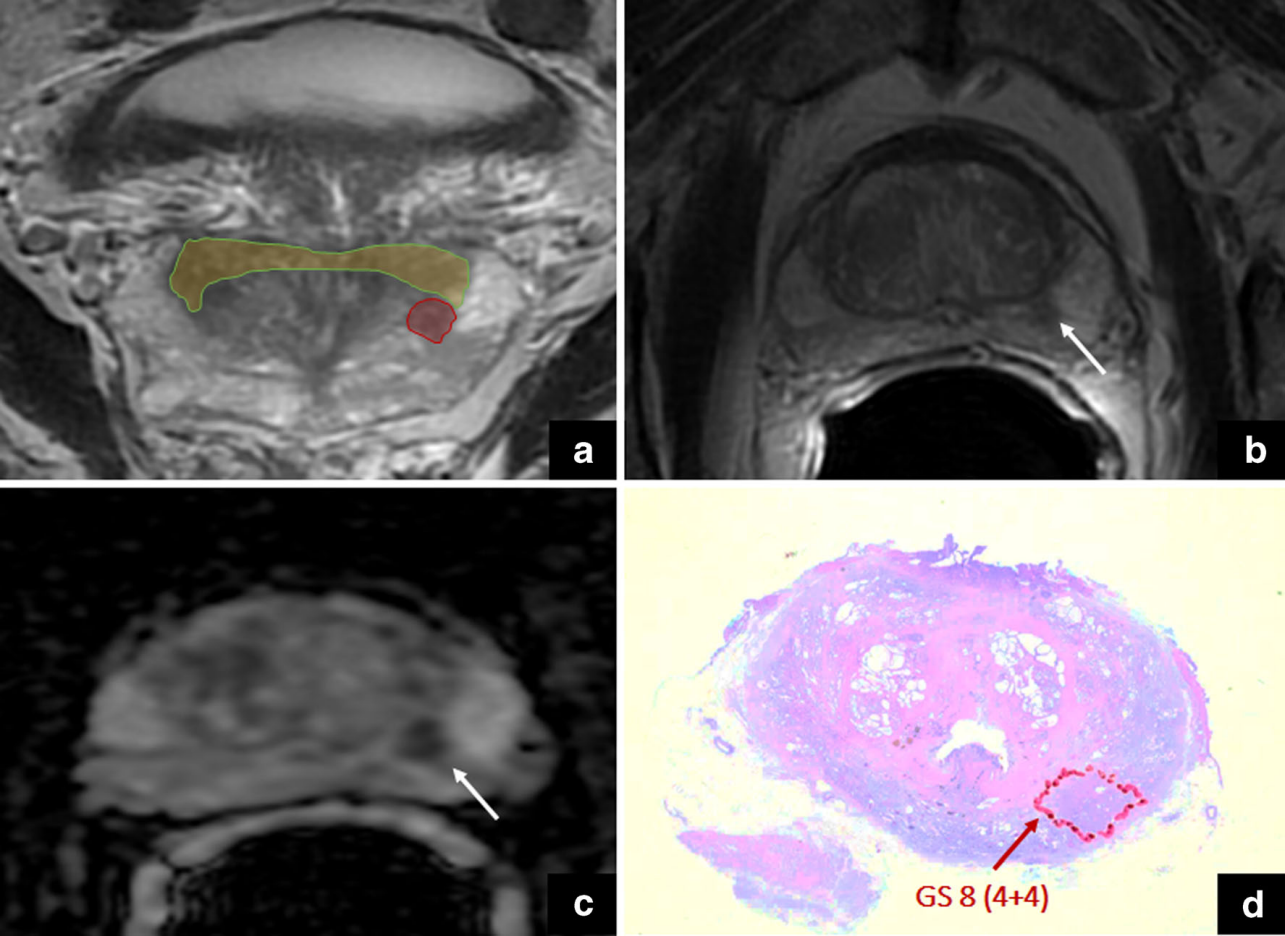
The yellow area is the *moustache sign*. The arrows show a focal, asymmetric area characterised by low signal intensity on coronal (**a**) and axial (**b**) T2-weighted imaging, together with marked restriction on DWI (**c**). These findings suggest a suspicious lesion in the left peripheral zone (cancer in *moustache sign*), and this was confirmed at final histology after radical prostatectomy (GS = Gleason score 4 + 4) (**d**)

This is a clear example where the radiologist could be initially misled by PI-RADS v.2 but the knowledge of both the anatomy and the pitfall assists in the detection of PCa (*cancer in moustache sign*) (Fig. 6).

#### Median posterior BPH proliferation (*teardrop sign*)

**Fig. 7:**
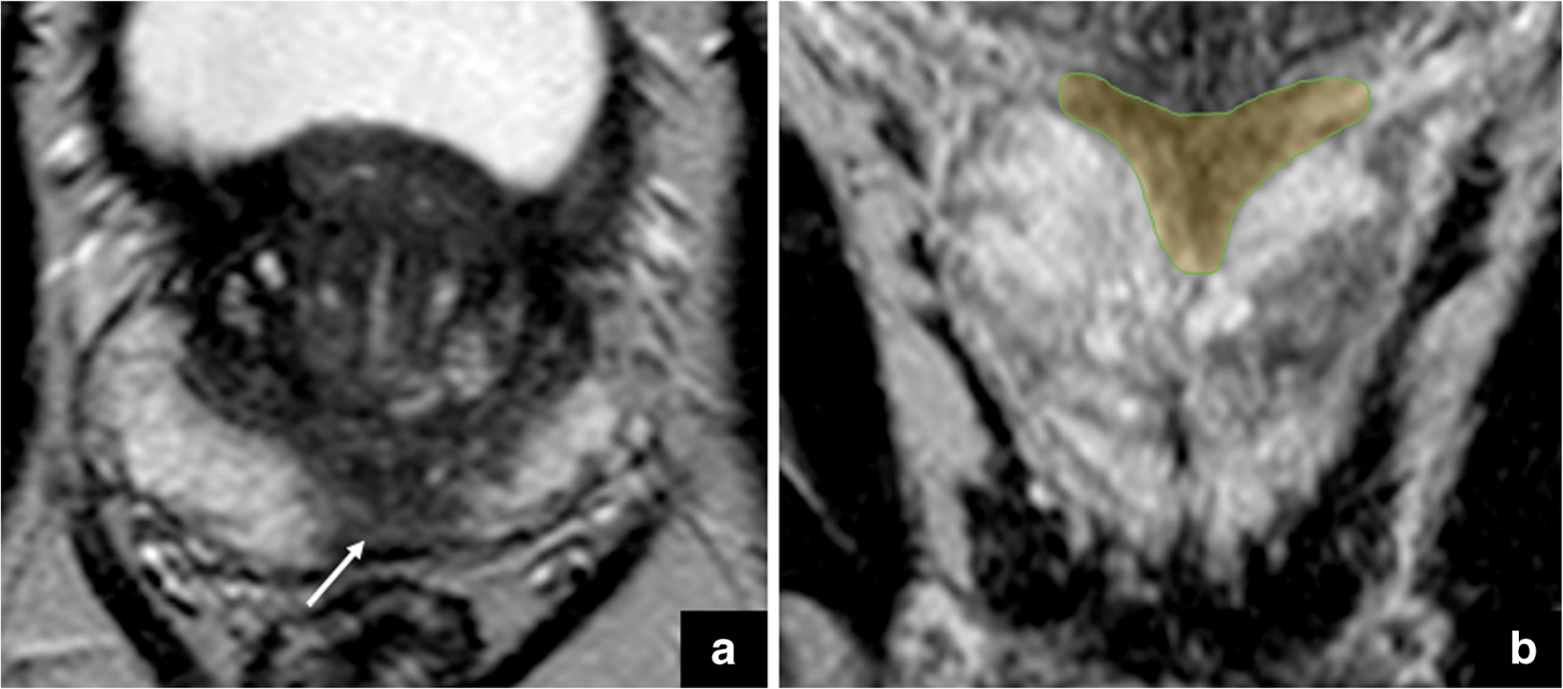
Axial T2-weighted image (**a**) that shows a hypointense area at the prostate base, in the peripheral zone. This aspect is a variant/extension of the *moustache sign*, in which the central zone is compressed between the transitional and peripheral zones, adopting a *teardrop* shape, as represented by the yellow area in the coronal T2-weighted image (**b**)

The presence of a focal/nodular, hypointense area at the middle third or at the base (adjacent to the ejaculatory ducts) of the PZ of the prostate could mimic cancer. This aspect is a variant/extension of the previously described *moustache sign*, in which the central zone is compressed between the TZ and PZ, adopting a *teardrop* shape (Fig. 7).

Here, the pitfall is related to a posterior bulging of the central zone *above* the *verumontanum*, between the TZ and the PZ (*teardrop*), and could show mild, restricted diffusion and focal contrast uptake. The low signal intensity of this finding on T2-WI is due to the hypertrophic tissue that follows the ejaculatory ducts before entering the prostatic urethra.

**Fig. 8:**
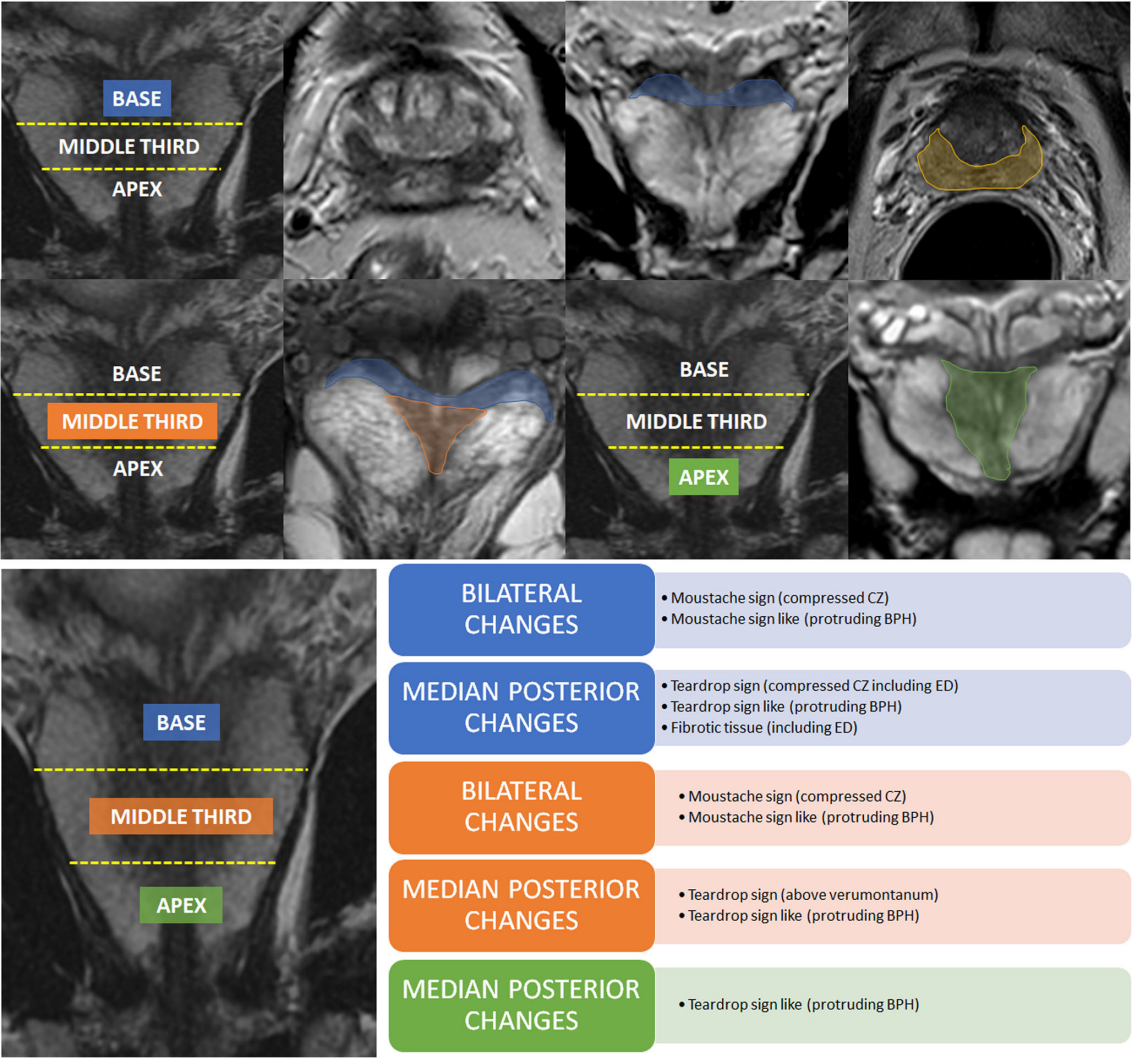
Summary of the different signs (*moustache* or *teardrop*) at different levels. CZ = central zone; BPH: benign prostatic hyperplasia; ED = ejaculatory ducts

**Fig. 9:**
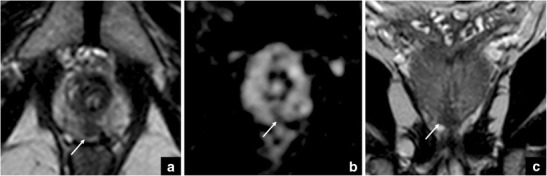
The arrows show a focal area with low-signal intensity on axial (**a**) and coronal (**c**) T2-weighted imaging, and restricted diffusion on DWI (**b**) at the prostate apex. The use of the coronal plane is very important to differentiate a pitfall (*teardrop*) from prostate cancer (*cancer in pitfall*)

It follows that the *verumontanum* plays a crucial role to distinguish between the compressed central zone and protruding BPH. If our finding lies *above* the *verumontanum*, this can be due to both a bulging central zone (*teardrop sign*) or a protruding BPH (*teardrop-like sign*), while only BPH will be present *below* the *verumontanum*. Figure 8 is a summary of these signs (*moustache* or *teardrop*) at different levels. According to PI-RADS v. 2, a potential score for this pitfall could be 3/5 for T2-WI, 3/5 for DWI and early enhancement (+) on DCE, suggesting the presence of clinically significant PCa, with an overall score of 4/5. We want to emphasise that the use of the coronal and/or sagittal plane is very important to demonstrate the continuity and symmetry of this area with the rest of the central portion of the gland, and it is crucial to differentiate a pitfall from PCa (*cancer in pitfall*) (Fig. 9). As already discussed for the *moustache sign*, the radiologist could be initially misled by PI-RADS v.2 but the knowledge of both the anatomy and the pitfall can assist in the detection of PCa in the presence of a reversed teardrop area.

An area that is not in continuity with the central portion of the prostate, characterised by marked low signal intensity on T2-WI (scored as 4/5) together with an early uptake of contrast (+) and a high grade of restriction on DWI (scored as 4/5) can be correctly classified as a highly-suspicious lesion.

**Fig. 10:**
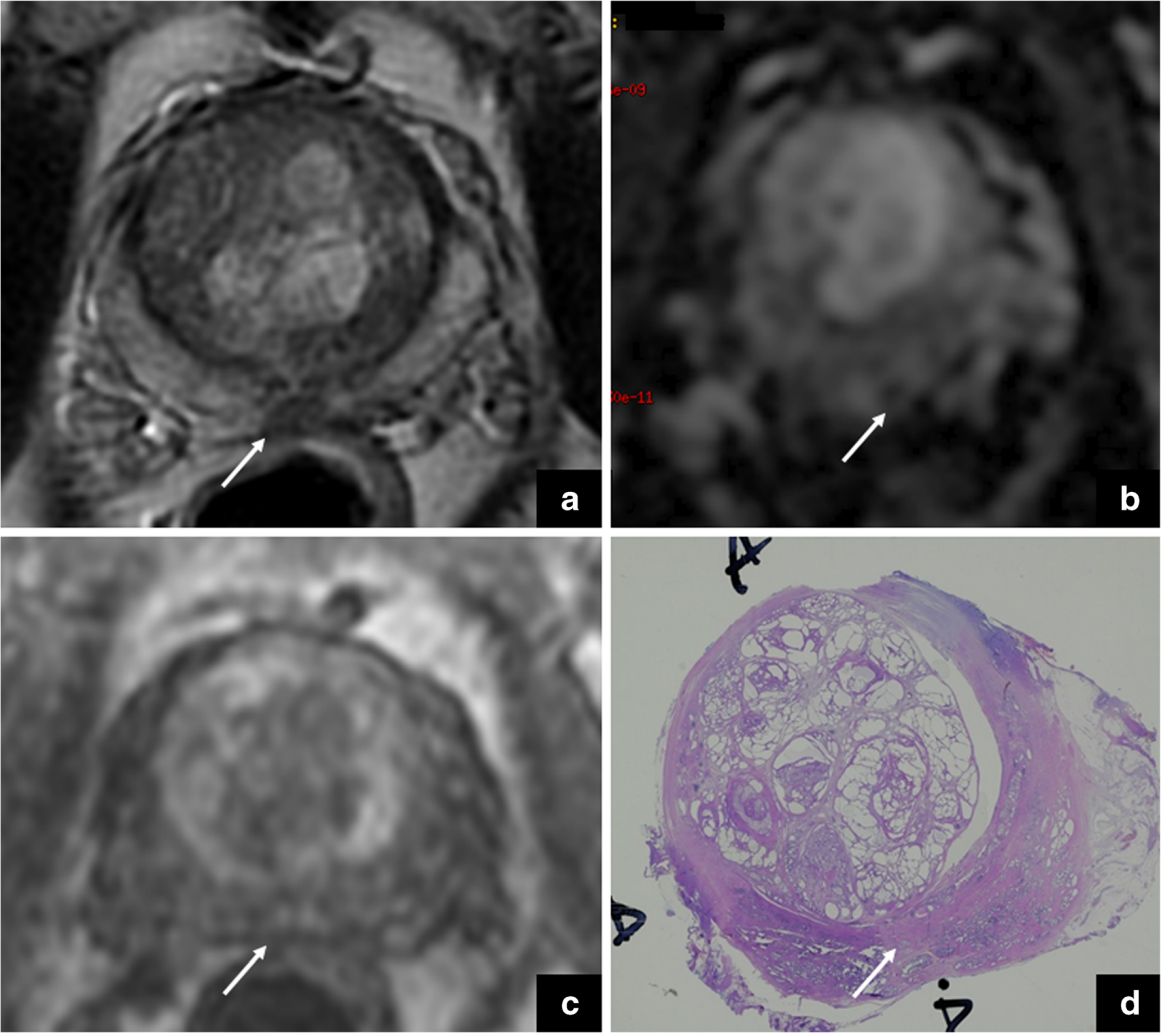
Axial T2-weighted image (**a**) that shows a focal area of low signal intensity adjacent to the ejaculatory ducts (white arrow). This corresponds to an area of restricted diffusion on the ADC map (**b**) and late enhancement on DCE imaging (**c**) and represents fibrosis, as confirmed at final histology after radical prostatectomy (**d**)

Again, it is very important to understand the mpMRI anatomy of the prostate to detect cancer in the context of a pitfall (median posterior BPH proliferation). Sometimes some fibrotic tissue can be seen adjacent to the ejaculatory ducts. This is a low-signal intensity area, with restricted diffusion but showing late enhancement on DCE imaging (i.e., there is no early contrast uptake like in PCa) (Fig. 10).

#### Ectopic BPH nodule

**Fig. 11:**
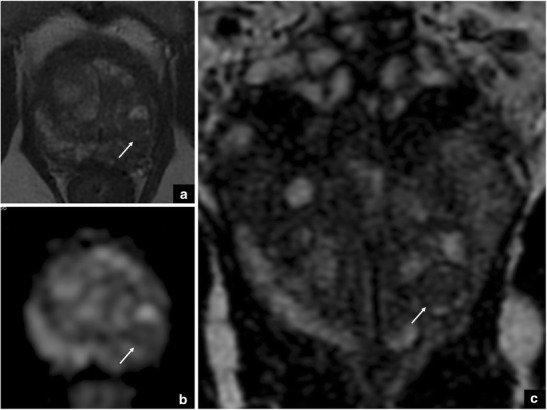
The arrows show a focal nodule bulging in the left peripheral zone, characterised by low signal intensity on axial (**a**) and coronal (**c**) T2-weighted imaging, with sharply defined margins, and restricted diffusion on the ADC map (**b**). The presence of tiny bright spots (corresponding to dilated acini) is consistent with a nodule of stromal benign prostatic hyperplasia (BPH), which may sometimes protrude from the central zone

The presence of an ectopic, focal peripheral nodule characterised by low signal intensity on T2-WI, with sharply defined margins, restricted diffusion and enhancement similar to the central portion of the hypertrophied TZ, could be erroneously interpreted as PCa in the PZ [12–13]. Moreover, the presence of the pseudocapsule along with tiny bright spots (corresponding to dilated acini) is consistent with a nodule of stromal BPH, which may sometimes protrude from the central zone. Not recognising an ectopic nodule of BPH in the PZ may lead to the use of DWI as dominant sequence in the PZ, grading this area as 4/5. Conversely, in the presence of an ectopic nodule, T2-WI can be used to score this zone, as this is the dominant sequence for the TZ. Such an approach would yield a 2/5 score and, therefore, downgrade the finding from a malignant lesion to a benign condition (Fig. 11).

However, as there are no established guidelines that suggest scoring a TZ lesion that protrudes into the PZ using the dominant sequence from the TZ (rather than PZ), we deem that in this scenario the experience of the radiologist and the knowledge of prostate anatomy and specific morphological features of BPH (e.g., regular capsule and margins) are more important than ever.

#### Prostatitis

This condition is usually caused by *E. coli* or Staphylococcus infections, and can ultimately result in the formation of an abscess.

On mpMRI, focal prostatitis can show an area of decreased signal on T2-WI (from nodular to band-like) in the PZ (adjacent to the capsule and infiltrating the periprostatic fat; hence, mimicking extracapsular extension – T3 stage) and increased perfusion on DCE (+), yielding a “false positive” finding. Additionally, the ADC map can be characterised by an area of low signal intensity.

**Fig. 12:**
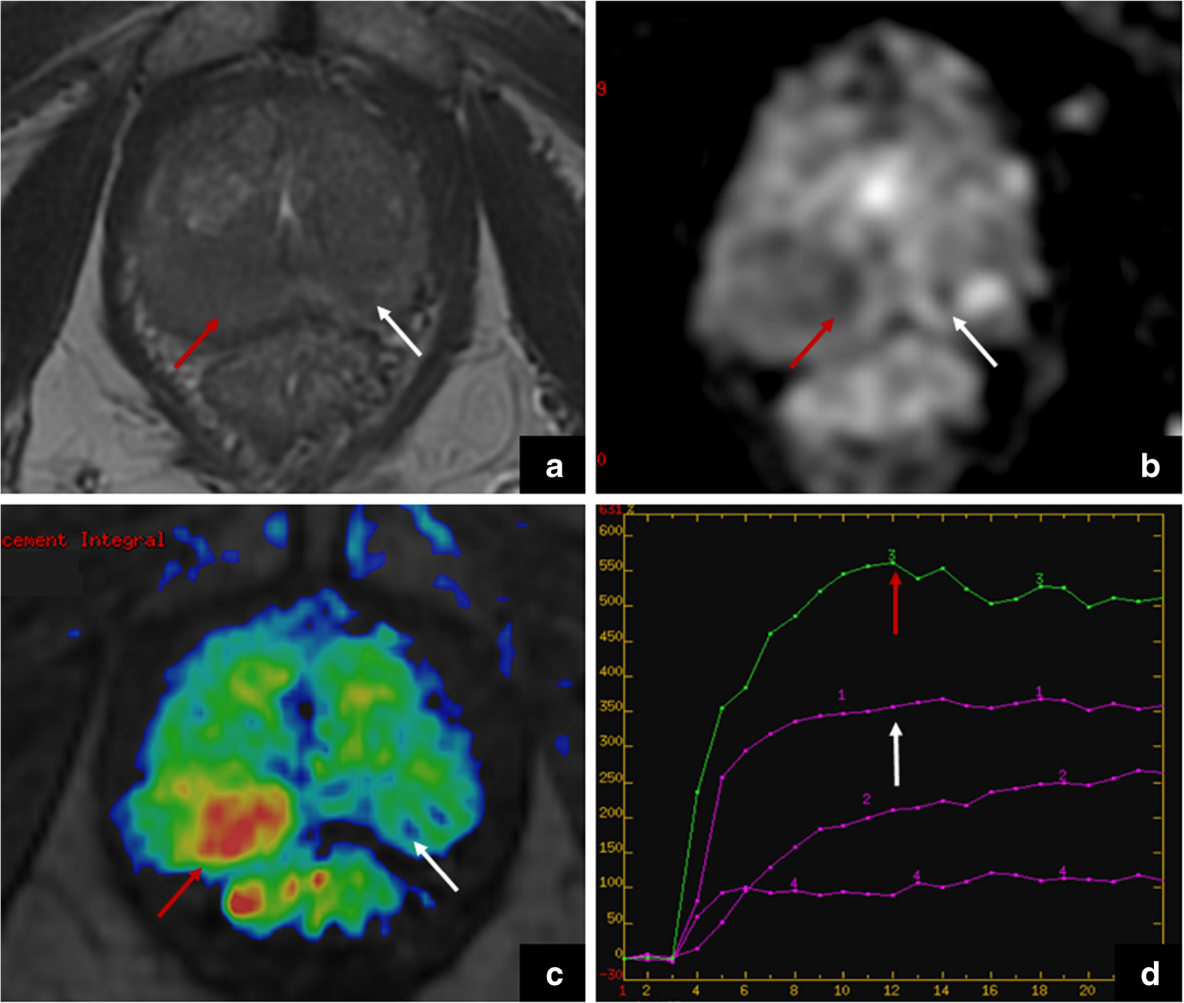
The red arrows show a focal area in the right peripheral zone with low-signal intensity on axial T2-weighted imaging (**a**), restricted diffusion in the ADC map (**b**), avid enhancement in the DCE study (**c**) and an early wash-in curve (**d**); these findings are consistent with prostate cancer (Gleason 3 + 3). The white arrows show a diffuse area of decreased signal in the left peripheral zone on T2-weighted imaging (**a**), and a mild restriction in the ADC map (**b**) and diffuse contrast uptake (**c** and **d**); these findings are consistent with prostatitis

According to PI-RADS v.2, the aforementioned findings can be scored at least 4/5, suggesting the presence of clinically significant PCa. However, the clinical history plays a crucial role in the diagnosis of focal prostatitis vs the presence of PCa (incidental finding), as this latter shows an earlier and more avid enhancement (*cancer in pitfall*) (Fig. 12).

Therefore, final histopathology is regarded as the only means to carry out an accurate diagnosis and to exclude the presence of tumour.

In addition, the radiologist should keep in mind that also granulomatous prostatitis can occur in the prostate. This usually presents as a firm nodule on digital rectal examination and elevated PSA, thus mimicking PCa. Although there can be different causes (instillation of intravesical bacille of Calmette-Guérin for bladder cancer, tuberculosis, surgical procedures), most cases are usually idiopathic [9].

#### Abscess vs cancer

In the PZ, it is possible to find a round-shaped region characterised by inhomogeneous, low-signal intensity on T2-WI, with a pseudocapsule (scored as 2/5), together with ring enhancement on DCE (+) and restriction on DWI (scored as 4/5).

**Fig. 13:**
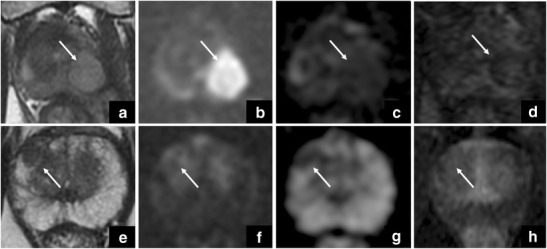
The first four images show a round-shaped area characterised by intermediate signal intensity on T2-weighted imaging (**a**), restricted diffusion on DWI (**b**) and in the ADC map (**c**), and ring enhancement (**d**). These findings, together with clinical history, orient towards the diagnosis of abscess. The other four images show a focal nodule in the right anterior peripheral zone, characterised by low signal intensity on axial T2-weighted imaging (**e**) with sharply defined margins and tiny bright spots, and restricted diffusion on DWI (**f**) and in the ADC map (**g**), and homogeneous enhancement on DCE imaging (**h**). These findings are consistent with an ectopic nodule of BPH

In this context, one might argue that PI-RADS v.2 is erroneously suggesting the presence of clinically significant PCa, but the specific ring enhancement together with the clinical history (e.g.. fever) can orient towards the correct diagnosis of abscess (Fig. 13).

#### Haemorrhage and other pitfalls

The presence of haemorrhage after prostate biopsy is relatively frequent. In fact, citrate is normally produced by the prostate for preserving the semen, but it is also an endogenous anticoagulant that can lead to protracted bleeding and non-coagulation of blood after biopsy. The latter may cause decreased T2 signal intensity that could mimic or obscure a suspicious area for PCa. Using a strict approach of PI-RADS v.2 guidelines, this should be grading as 4/5 on T2 and 4/5 on DWI, with a low ADC. However, the pre-contrast T1-WI can help to differentiate this area from a suspicious focus of PCa, as it shows a mild hyperintense signal due to the products from the haemoglobin degradation. This will be also supported by the corresponding hypointense signal in the post-contrast subtraction imaging.

**Fig. 14:**
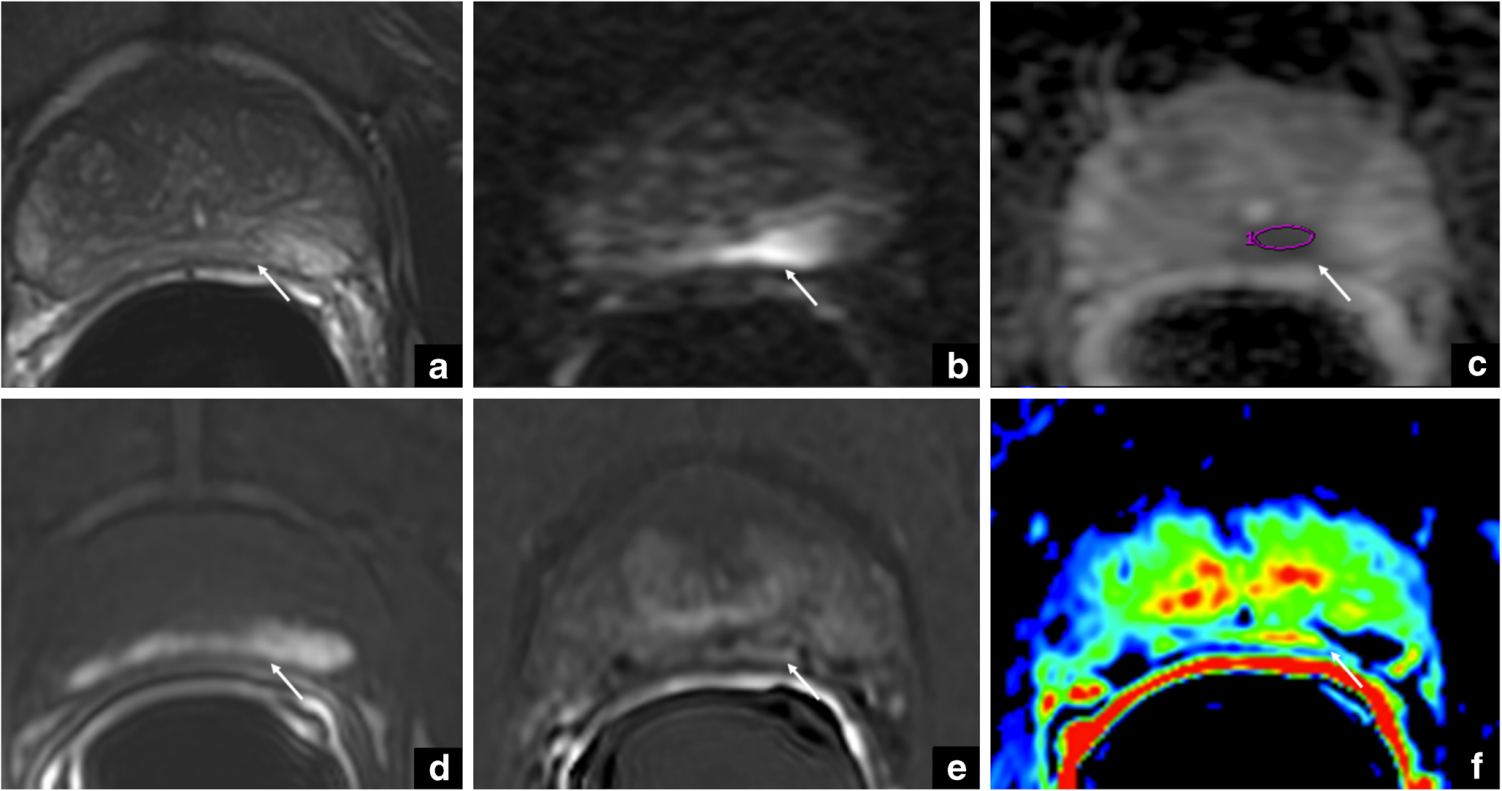
The arrows show an area of mild, low-signal intensity on T2-weighted imaging (**a**), with restricted diffusion on DWI (**b**) and in the ADC map (**c**). This corresponds to a hyperintense area on pre-contrast T1-weighted imaging (**d**), which is consistent with the products from the haemoglobin degradation after biopsy, as also supported by the post-contrast subtraction imaging (**e**) and in the colour DCE map (**f**). DCE studies have been obtained by gradient-echo sequences (TR: 4,5 ms; TE: 1,5 ms; flip angle: 15°; Average: 4; slice thickness: 2 mm; Matrix: 320 × 320; Scan Time: 3.13 min), using a body-weight adjusted intravenous bolus of gadobutrol (Gadovist, 1 mmol/mL; Bayer Schering Pharma, Berlin, Germany). DWI parameters were: TR ≥ 3000 ms; TE ≤ 90 ms; slice thickness ≤ 4 mm, no gap; field of view:160–220 mm; in plane dimension ≤ 2.5 mm phase and frequency; b values for DWI were 0–500- and values ranging between 1000 and 3000 s/mm^2^. For ADC maps, if only two b values can be acquired, it is preferred that the lowest b value should be set at 50–100 s/mm^2^ and the highest should be 800–1000 s/mm^2^

When the clinical question is detecting PCa, it is of utmost importance to impose a delay after biopsy, to allow time for re-absorption of blood products (approximately 4–8 weeks) (Fig. 14). The delay could be different based on why mpMRI is done (i.e., detection vs staging). As staging is more dependent on T2-WI, delay after biopsy for staging is more desirable, while for detection it might not be necessary.

Focal atrophy - particularly the post-atrophic hyperplastic subtype - may mimic PCa on mpMRI due to the glandular crowding and complex architecture. Causes of atrophy include inflammation, irradiation, antiandrogen therapy, and chronic ischaemia from local arteriosclerosis. Focal atrophy occurs more frequently in the PZ and appears as a focal or geographical area of low T2 signal intensity on mpMRI, with both moderate diffusion restriction and enhancement. The degree of restriction and tissue enhancement are usually less marked than PCa.

Necrosis can be seen after the resolution of the abscess and florid inflammatory changes from an infectious prostatitis, or after focal therapy. Necrosis shows low T2 signal intensity and diffusion restriction, due to the coagulative state characterised by reduced water movement, as well as by the adjacent inflammatory infiltrate and atrophy. There is also no enhancement. Together, these features suggest the presence of necrosis and fibrosis on mpMRI.

Calcification is due to concreted prostatic secretions, calcified corpora amylacea and phleboliths in the periprostatic venous plexus. Calcifications show low signal intensity on T2-WI and ADC images, together with no enhancement and a persistent, marked low signal intensity on DWI at all *b* values.

All the aforementioned pitfalls (focal atrophy, necrosis and calcifications) have specific features that help to distinguish them from PCa when applying PI-RADS v. 2 (e.g., no enhancement or less restriction on DWI).

## Pitfalls related to technical and physiological artefacts

The use of an endorectal coil in addition to the surface coil improves the signal-to-noise ratio and the spatial resolution both at 1.5 and 3 T. On the contrary, patient or bowel movements during image acquisition may cause repetitive circular artefacts along the boundaries of the endorectal coil. These artefacts can be minimised by rectal emptying and by the administration of a spasmolytic drug prior to the examination.

According to PI-RADS v. 2, an area of homogeneous low-signal intensity on T2-WI in the PZ, showing restricted diffusion on DWI and focal enhancement (+) can be scored as 4/5, suggesting the presence of clinically significant PCa. However, the use of a single surface coil could erroneously suggest extracapsular extension (T3 stage) because of the lower resolution. In this case, the application of PI-RADS v. 2 together with the use of the endorectal coil could help to rule out capsular involvement (T2 stage), thanks to the increased resolution.

**Fig. 15:**
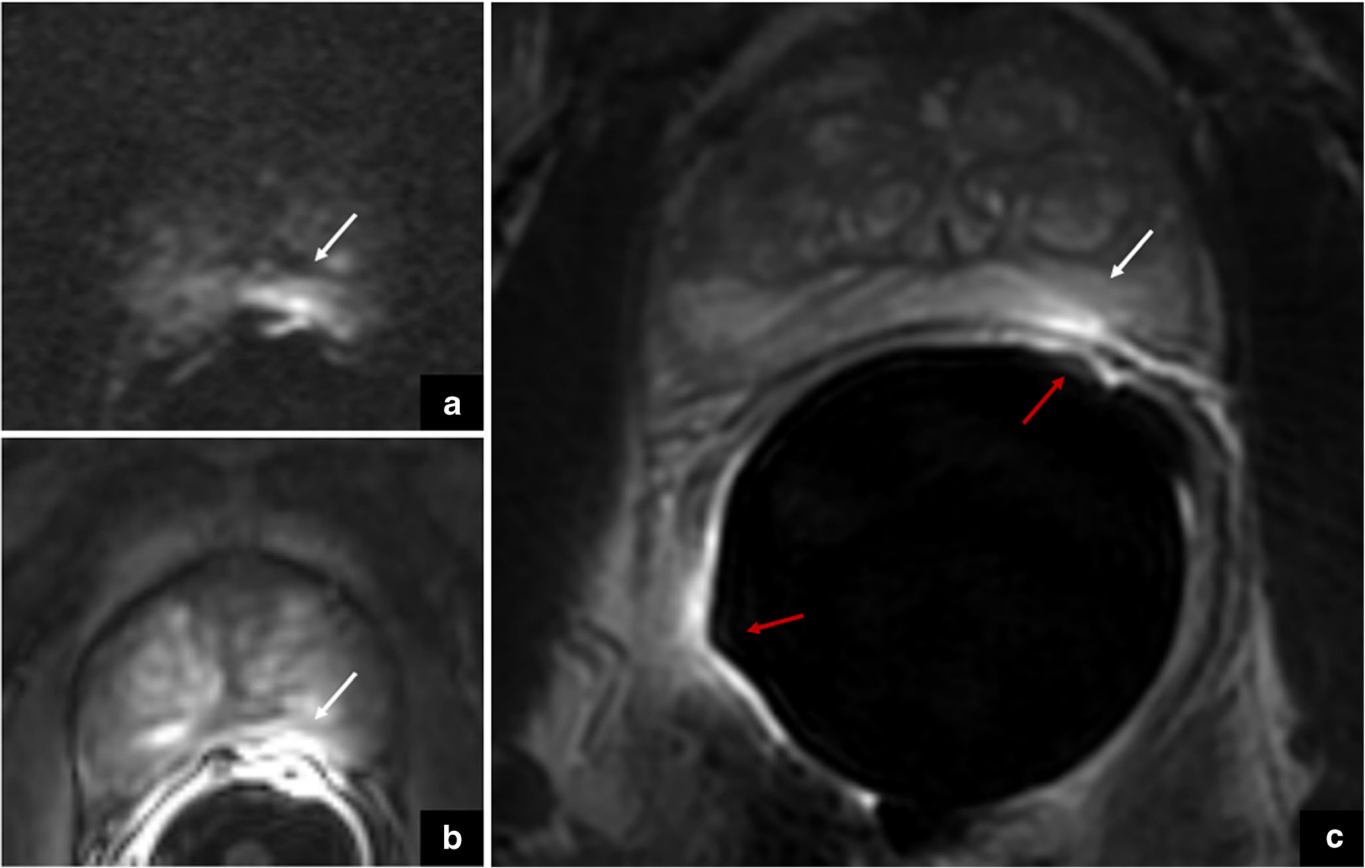
The white arrows show an area mimicking restricted diffusion on DWI (**a**) and focal enhancement on DCE imaging (**b**) in the left peripheral zone, very close to the prostatic capsule and adjacent to the endorectal coil surface. This finding could be erroneously interpreted as a lesion, but the axial T2-weighted image (**c**) does not show this artefact, and therefore prostate cancer can be ruled out. Red arrows show the coil surface

The endorectal coil should be positioned correctly, in order to avoid the risk of incurring potential diagnostic pitfalls that could mimic the presence of PCa. The correct position of the coil is in a plane perpendicular to the left-right phase encoding direction. If the coil is not positioned properly, it is possible to see a focal area of enhancement (+) with restricted diffusion (scored 4/5 according to PI-RADS v. 2) adjacent to the coil surface. This finding could be erroneously interpreted as a lesion (Fig. 15), but the ADC maps and T2-WI do not show this artefact, and therefore PCa can be ruled out.

To sum up, there are different artefacts that can mimic the presence of PCa and which the radiologist should be aware of. These include the presence of gas in the rectum, and the interpretation of ADC maps with low-value pixels, as these latter can show the same dark signal as fat, creating problems for lesions along the capsule. As far as the use of spasmolytic agents is concerned, the radiologist should bear in mind that many patients undergoing mpMRI will have a large prostate due to BPH (with related urinary problems). Therefore, these medications should be administered carefully and preferably before positioning the patient on the mpMRI table. Glucagon should be chosen in patients with urinary retention.

## Conclusions

Since its introduction, mpMRI of the prostate has been changing the management of suspicious PCa, especially in men with non-specific high PSA, where the detection of clinically significant PCa has been shown to be more accurate than standard TRUS biopsy [14]. Despite the standardised attempts to report mpMRI (PI-RADS v 2) [7], some para-physiologic appearances of the prostate gland can mimic cancer. As there are no established guidelines that suggest scoring a TZ lesion that protrudes into the PZ using the dominant sequence from the TZ (rather than PZ), we deem that in this scenario the experience of the radiologist and the knowledge of prostate anatomy and specific morphological features of BPH (e.g., regular capsule and margins) are more important than ever. Moreover, we believe that the radiologist should be aware of clinical data such as the exposure to antiandrogen therapy for BPH, as this could affect the conspicuity of tumours in the TZ as well as on DWI. The radiologist should also keep in mind that other sequences (e.g., DWI) can be of great help while reporting prostate mpMRI, if there are some doubts on T2-WI, as suggested by the PI-RADS v. 2 guidelines [15–21]. Although these guidelines use DWI to upgrade some PI-RADS 3 lesions in the TZ to PI-RADS 4, DWI alone is nonetheless a sensitive sign for detection of tumours even in the TZ [22–23]. Rosenkrantz and colleagues [22] reported that the incorporation of DWI and ADC maps (b value:1000 s/mm^2^) significantly improves the sensitivity for TZ tumours compared with T2-WI alone. The main reason is the diffuse background heterogeneity and the presence of multiple nodules in the TZ, which make tumours harder to identify on T2-WI. As known, DWI investigates the movement of water molecules within tissues and reflects changes in cellularity; thus, it provides complementary information that may help depict lesions not initially visible on T2-WI, leading to improved sensitivity.

In conclusion, we recommend that any radiologist involved in prostate mpMRI be fully aware of the pitfalls mentioned in this pictorial report, in order to avoid underestimation and overestimation of PCa detection. We also deem that this manuscript gives a repertoire of potential solutions for the improvement of the future PI-RADS guidelines.
